# Resilience in maize production for food security: Evaluating the role of climate-related abiotic stress in Pakistan

**DOI:** 10.1016/j.heliyon.2023.e22140

**Published:** 2023-11-10

**Authors:** Muhammad Rizwanullah, Anhua Yang, Muhammad Nasrullah, Xue Zhou, Atif Rahim

**Affiliations:** a School of Public Administration, Xiangtan University, PR China. 411105; b South Asia Research Centre Xiangtan University, PR China. 411105; c Business School of Xiangtan University, PR China. 411105

**Keywords:** Maize production, Climate change, Food security, Technology level, Sustainable agriculture development

## Abstract

The primary purpose of this research is to examine the impact of climate change on maize production in Pakistan. This research studied the impact of climate change on maize production in Pakistan from 1990 to 2020 using the Auto Regressive Distributed Lag (ARDL) technique and draws implications for the future of Pakistan's sustainable agricultural industry. According to ARDL's short-run and long-run analyses, variables such as average temperature (AVEGTP), carbon dioxide (CO2), precipitation (PRPT), and tube well irrigation (TWL) all have a significant short-run and long-run impact on maize yield at the 1 %, 5 %, and 10 % significance levels. The estimated findings were also affirmed through FMOLS and DOLS. The study's key findings indicated that variables such as average temperature, carbon dioxide, precipitation, and tube well irrigation had significant short-run and long-run impacts on maize yield. Climate change's impacts on maize yield underline the crucial need for action to address this global issue and ensure agriculture's future. A recent study has emphasized the significant impact of climate change on Pakistan's maize production, stressing the importance of addressing this global issue for food security. The study recommends selecting crop varieties and managing fertilizer applications based on projected climate change to mitigate the impending crisis. Policymakers can use the study's findings as valuable insights to formulate effective policies that ensure the resilience and sustainability of Pakistan's agricultural industry.

## 1 Introduction

### 1.1 Background of studies

Over the previous several decades, global warming has been worse. Unfavorable conditions for farming are becoming more prevalent and worse due to global warming [1]. Empirical evidence shows that climate change significantly harms agricultural productivity [2]. Hunger and malnutrition may be increased by stresses on food production, such as expanding disaster zones and declining food output. As a result, many nations are having more open discussions about these problems than ever before [3]. Improving Irrigation Systems [4], developing a "climate-smart food system" [5], and farmer education on how to adapt to climate change are just a few examples of the strategies that researchers argue for different countries to discover swiftly to mitigate climate change's detrimental impacts.

Humanity faces a significant threat from climate change and its repercussions in the 21st century. It is a global phenomenon that devastates ecosystems, economies, and societies everywhere, especially in the world's poorest regions [6]. Regarding agriculture, Pakistan is one of the countries most in danger due to climate change [7]. Pakistan's agricultural sector contributes over 20 % to GDP, whereas 40 % of the population is employed in manufacturing. Pakistan grows more maize than any other grain crop, second only to wheat. However, the effects of climate change on Pakistan's maize output are uncertain [8]. Water shortage during the growth phase may be caused by several variables, including changes in precipitation patterns (both geographically and temporally) and direct implications on the agricultural water process [9]. The quantity of water lost through evaporation and transpiration exhibits a significant degree of sensitivity to alterations in temperature [10]. Temperature increases that persist throughout the growing season have been shown to affect agricultural yields [11]. On the other hand, research shows that short periods of increased temperature during critical growth phases might increase infertility and decrease agricultural yields [12].

Different earlier studies have examined the degree of clarity surrounding the impacts of climate change on agriculture in emerging economies [13]. Due to a lack of sophisticated infrastructure, low-income nations are especially at risk from climate change [14]. Because of inadequate adaptation methods, changing temperatures and rainfall levels reduce crop production in low-income countries. The vulnerability in question has emerged due to the adverse impacts of recent flooding events and prolonged periods of drought, particularly during the 20th century [15].

Concerns about global warming's impact on Europe's neighbors have increased. Temperature and precipitation trends in Northern Europe have improved, while those in Southern and Eastern Europe have declined [16]. The recent rainfall was beneficial for farmers in South Africa. South Africa received timely summer and winter precipitation [17]. The magnitude of the climate change effects in Bhutan makes it more important to evaluate how much the country's agricultural sector is tied to the seasonality of the monsoon and temperature fluctuations. The sector might anticipate more losses because of minor changes in the frequency and severity of weather extremes. The adverse effects of these factors are seen most acutely in agriculture and food security. The global catastrophe sequence of climatic changes has significantly impacted India's agricultural economy. Warmer temperatures have been demonstrated to increase rice yields while decreasing wheat yields [18]. Climate change has already had detrimental effects on agriculture in Bangladesh, such as reducing crop yields by hundreds of tons annually. The agricultural sector has experienced significant hindrances due to various factors, including rising sea levels, heightened frequency and intensity of floods, and the formidable monsoon [19].

**Fig. 1:**
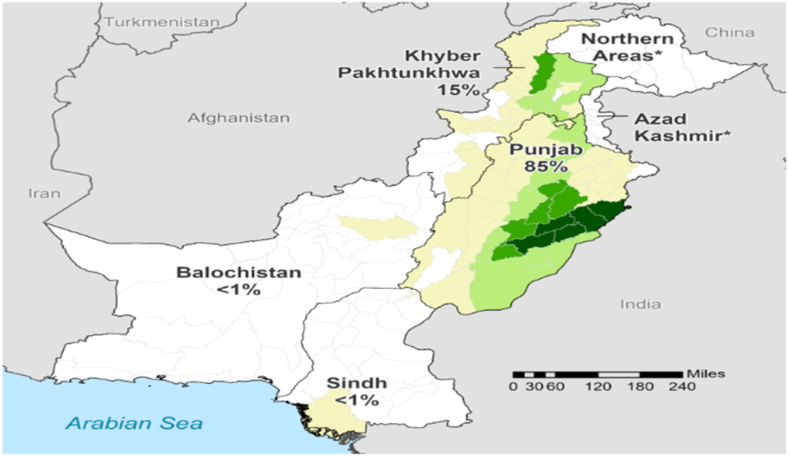
Pakistan maize production.

Pakistan has a substantial longitudinal reach from the Arabian Sea to the Himalayas in the north. There is a transition zone between the subtropical and temperate zones where the place is located [20]. Fig. 1 shows that the maize production province-wise and in this significant production of maize in Punjab is 85 % and Balochistan and Sindh are 1 % [21]. Major crops like rice, cotton, wheat, sugarcane, anise, and more, like masoor, mash, mung, potato, chilis, and onion, make up Pakistan's agriculture sector. Pakistan's two main growing seasons are Kharif and Rabi. The Kharif planting and harvesting seasons are April to June and October to December. The Rabi season begins in the fall (Oct. to Dec.)and ends in the spring (April to May) [22].

**Fig. 2:**
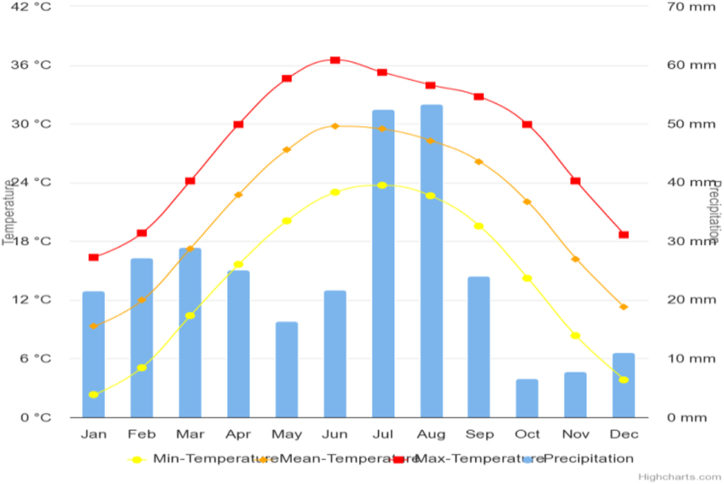
(Monthly climatology of min, Max, mean temperature and precipitation of Pakistan).

In Pakistan, climate change is becoming a significant risk to the agricultural sector. It is vulnerable to the effects of erratic weather [23]. Fig. 2 shows the monthly climatology of minimum, maximum, and mean temperature and precipitation in Pakistan from the World Bank [24]. It is anticipated that the production capacity will experience a significant decline in the forthcoming period. The effects of climate change on agricultural practices have been extensively studied in prior research [25]. According to Ref. [26], even a modest increase in precipitation benefits wheat yields. An augmentation in the average lowest temperature during the sowing stage is positively correlated with an expansion in production capacity.

In contrast, an escalation in the average maximum temperature during the mature stages is associated with reduced production output. The authors [27] evaluated the susceptibility of wheat to temperature increases in both the short and long term. While it was discovered that higher temperatures were beneficial to rice production at first, it was later discovered that temperatures above a certain optimal level were detrimental to rice production. It was determined that rainfall had a detrimental impact on rice yield. Rising temperatures also had a negative impact on sugarcane harvests [28].In Pakistan's cropping system, maize is a significant cereal crop. It has three major applications: human food, chicken feed, and livestock fodder. One of the highest-yielding cereal crops is maize. Pakistan likewise places a high value on maize. After wheat and rice, it is the third most common cereal worldwide [29]. Pakistan is a significant contributor to global maize production, so this research aims to assess the effects of climate change on the country's maize production, evaluate the usage and adoption of technology by maize farmers, and propose policy derivatives to mitigate the long-term and short-term consequences of this period. The study is organized as follows: introduction, literature review, methodology, results, discussion, and conclusion.

### 1.2 Objectives

The research objectives are.

• To examine climate change's actual and potential effects on maize production in Pakistan.
• To evaluate the role of technology in maize production.
• Recommend some possible policies based on the findings to the government.

To determine the uniqueness of this research issue, a well-designed study with reliable methods and data is needed. The research should review the literature to address gaps and advance past work. Due to climate change, Pakistan may profit from this research by increasing maize yield and food security. Understanding how Pakistani communities adapt to climate-related maize yield changes and build resilience is essential to grassroots climate adaptation. Pakistani agricultural resilience programs can benefit from studying maize production and climate. Understanding the relationship between climate and maize output could inspire new policies.

## 2 Literature Review

### 2.1 Theoretical foundation

#### 2.1.1 Resilience theory

Resilience theory is a comprehensive framework that helps ecological and agricultural systems recover from various disturbances or stresses [30]. This study focuses on the impact of climate change and other factors on the maize production system in Pakistan. According to resilience theory, a system's ability to recover quickly from disturbances is improved by having several independent components. We applied this theory to Pakistan's maize production system to identify strategies to enhance its resilience. These strategies include promoting crop diversity and implementing climate-smart agricultural practices. Ultimately, this research can help develop more resilient agricultural practices that can withstand the effects of climate change.

#### 2.1.2 Food security framework

Theoretical foundations for food security should involve understanding food supplies' access, availability, use, and security [31,32]. This model provides a broader understanding of Pakistan's food security and the significance of resilient maize production. By integrating food security concepts into the theoretical basis, policymakers and researchers can comprehensively understand the factors contributing to a nation's food security. This model focuses on the access, availability, and use of food supplies and highlights the importance of ensuring their security. It is essential to comprehend the role of resilient maize production in Pakistan's food security to develop effective strategies to address potential challenges or vulnerabilities in the future.

#### 2.1.3 Agroecological system theory

The concept of agroecological systems examines how agricultural and environmental factors interact [33,34]. It can be applied to understand better how maize production is affected by climate-related stressors and how resilient practices can be incorporated to make these systems more robust. By recognizing the connection between agricultural and environmental factors, the theory of agroecological systems offers valuable insights into the impact of climate-related stressors, such as drought or extreme temperatures, on maize production. These stressors can have a significant impact on crop yields and productivity. Therefore, implementing resilient practices based on agroecological principles can help mitigate these effects and increase the resilience of maize production systems in the face of changing climate conditions.

### 2.2 Empirical evidence

As global average temperatures have steadily climbed, they have set off a chain reaction of extreme weather that has wreaked havoc on farming and the food supply [35]. This research estimates the effect of temperature, Precipitation, and sunshine hours on maize output using the Transcendental Logarithmic Production model, using panel data from the National Rural Fixed Observation Point in China from 2009 to 2018, about 3150 small farmers engaged in maize planting [36]. Considering the variations in regional climates, this study [37,38] also analyzes the expansion potential of China's five main maize-producing regions. The findings show that higher temperatures, precipitation, and rainfall increase maize yields, but less sunlight reduces expansion. The five main areas for maize production are being hit differently by climate change, necessitating regionally specific adaptation measures [39]. Other significant factors in maize output are the area devoted to cultivation, the number of workers employed, and the total investment amount. Based on these results, according to Ref. [40], to encourage sustainable agricultural production, specific measures can be taken. Improve the accuracy of weather forecasts for major maize-growing regions; optimize agricultural output structure and growing structure considering local resources; launch watering and water preservation projects; and expand access to training programs that emphasize adaptive behavior in response to climate change.

According to researchers [41,42], tools for predicting future climate can be informed by changes in atmospheric CO2 concentration, temperature, and precipitation. Climate change (CC) is how these instruments change from their historical baselines due to present and future situations beyond human control [43]. Like all other life forms, crops can profit from or suffer from climatic shifts [44]. It is debatable whether or not the widespread cultivation of maize has had a net good or bad effect on Pakistan's agricultural sector [45]. Using vector autoregression models and time series data from 1980 to 2013, this study examined the potential consequences of global warming on Pakistan's maize yield [39]. The study's findings predicted that by 2030, a 6 % drop in annual maize yield will result from higher-than-average temperatures. Until 2021, a 9 % increase in maize yield can be attributed to the average minimum temperature. Maize crop production will also benefit from increased overall rainfall [46]. In 2030, compared to 1980 (the base year), maize output will have increased by 2 %. Water availability during the crop's growth stages and fertilizer use (now or in the future) will unquestionably boost output [47]. The government should make adaptation alternatives, notably for growing maize, more accessible to farmers to mitigate the consequences of the changing climate on agriculture. This looming crisis can be mitigated by selecting crop varieties and managing fertilizer applications in light of projected climate change [48].

Climate change has the potential to significantly reduce the profitability, productivity, and sustainability of agricultural output [49]. Few studies have examined how changes in climate affect maize production in Serbia's agricultural ecosystem [50]. The study aims to analyze how climatic changes through time and place affect maize cultivation in two regions of the Republic of Serbia. Central Serbia and Vojvodina's climate parameters (temperature, precipitation, sunshine, and humidity) were analyzed using data from Kragujevac and Sombor meteorological stations for two 30-year periods (1961–1990 and 1991–2020) [51]. The observed climatic parameters were analyzed using a correlation analysis from 2004 to 2020 to see whether and to what degree they are connected to maize production. Kragujevac saw an increase of 0.046 °C and Sombor of 0.05 °C in annual average temperatures between 1991 and 2020, whereas Sombor saw an increase of 5.01 h in yearly average sunlight. Humidity decreased by an average of 1.3 % points per year in Kragujevac and by 3.4 % points per year in Sombor between 1961 and 1990 [52]. Corn yields were considerably lower in years with severe heat and a lack of precipitation. Evidence from these studies shows that global warming impacts maize output, calling for more climate-resilient maize hybrids and varieties as well as improved agro-technical solutions [53]. The global food supply may be in jeopardy due to rising temperatures, altered precipitation patterns, the proliferation of extreme weather events, and the release of more greenhouse gases [54]. The increased frequency of extreme climate events causes more significant fluctuations in agricultural output [55]. For the anticipated period 2021–2050 and 2051–2080 under different Representative Concentration Pathway (RCP) 2.6, 4.6, 6.0, and 8.6 W/m2, the effect of climate change on yields of maize and characteristics was evaluated using the CERES-maize model [56]. The results were compared to the baseline scenario of 1982–2012 for eastern India. A 10.58% point drop in output between 2021 and 2050, a 14.80% point drop between 2051 and 2080, and a 23.39% point drop between 2051 and 2080 was found for irrigated circumstances, relative to the baseline period of 1982–2012. In rain-fed conditions, yield changes were reported at 10.55 %, 9.20 %, 8.13 %, and 7.47 % from 2021 to 2050, and at 10.63 %, 6.65 %, 7.47 %, and 4.31 % from 2051 to 2080, respectively [57]. The study found that grain yield decreased from 2051 to 2080 under irrigated conditions compared to the baseline yield. However, it increased from 2021 to 50 and 2051 to 80 under rain-fed conditions, indicating that the positive effect of rainfall on crop yield outweighed the negative effect of temperature [58].

Changes in climate, such as higher average temperatures and more frequent and severe weather events, are a big worry for farmers in Pakistan [59]. Pakistan's food security is critically dependent on the maize crop, which is very susceptible to the effects of climate change [60]. This study aims to review previous work on the issue of climate change's effects on Pakistan's maize production and the consequences this may have for agriculture's long-term viability in the face of unpredictable weather patterns. According to empirical studies, Climate change is already negatively impacting Pakistan's maize harvest [61]. As temperatures rose and rainfall patterns changed, the largest maize-growing province, Punjab, saw its yields diminish. Growing seasons for maize are getting shorter due to rising temperatures, which harms crop yields, as observed by Ref. [62]. Climate change is predicted to exacerbate existing problems with crop yields in Pakistan due to rising temperatures and more volatile weather patterns [63]. However, more study is needed to determine how carbon dioxide exacerbates climate change's effects on maize yields [64]. This literature review aims [65] to examine the existing data on how CO2 affects maize output in Pakistan against the backdrop of global warming. According to an empirical study, there will be both beneficial and detrimental consequences of increased atmospheric CO2 levels on maize output [66]. On the one hand, increased CO2 levels can increase maize production by stimulating photosynthesis and growth [67]. Research by Ref. [68] found that a 50 % rise in CO2 concentrations led to a 23 % increase in maize yields in China. Scientists have discovered that the negative consequences of climate change may cancel out the positive effects of CO2 on maize output [69]. found that increasing CO2 levels increased maize yields in Pakistan, but drought stress reduced such benefits.

According to the researcher [70], climate change threatens world food supplies by affecting agricultural production. China is the ^second^ largest producer and consumer of maize [71]. Understanding how climate change affects maize production can guide national and international economic and political policies [72]. Since panel models cannot identify group-wise heteroscedasticity, cross-sectional correlation, and autocorrelation, the researcher [73] used the feasible generalized least square (FGLS) model to assess the impact of climate change on Chinese maize yields from 1979 to 2016. China's maize harvest suffered from 1979 to 2016 temperature increases. Maize yield fell by 1.7 % every 1 °C, or 5.19 kg 667 m-2. The agricultural production barely changed due to the minor increase in rainfall. Maize production increased by only 0.043 kg 667 m-2 (0.014 %) for each millimeter of rainfall [74]. Climate change is especially harmful to maize production in southern China. A 1 °C temperature increase in this area reduced maize output by 7.49 kg 667m-2, while it had no effect in northern China. Southern China yields 0.013 kg 667 m-2 for every 1 mm of precipitation, while northern China yields 0.066 kg 667 m-2. The temperature had a smaller marginal influence in southern and northern China during 1990–2016 than from 1979 to 2016 [75].

This study [76] examines how research and development spillover and irrigation water utilization impact Pakistani agricultural output. In the face of water constraints, irrigated technology, internal and foreign R&D shocks, human resources, agricultural employment, and land-determined agricultural productivity [77]. We gather yearly time series data from 1973 through 2020 for these purposes. Knowledge spillover and water resource efficiency affect Pakistani agricultural productivity using the Autoregressive Distributed Lag (ARDL) model. The findings show that international and local R&D spillovers have benefited Pakistani agriculture productivity. External R&D spillovers improved agriculture productivity more than expected. Pakistan's agricultural output rose due to water technology, according to estimates. Human capital and interaction term results are substantial and unfavorable, demonstrating that labour in agriculture needs to be more skilled at learning from outside sources [78].

The study's [79] goal is to examine how CO2 emissions contribute to climate change and how formal credit affects AP in Pakistan. Tractors (TRs), tube wells (TWs), energy consumption (EC), and labour force (LF) are all included in the study's technical aspects. The years 1981–2017 were used to analyze the yearly data. Cointegration, also of the underlying variables, was investigated using an autoregressive distributed lag (ARDL) approach, and the direction of causality was assessed using the Granger causality test within the framework of the model for vector error correction (VECM). Organizational financing, atmospheric CO2 emissions, agricultural productivity, energy consumption, workforce magnitude, and technologies (including tractors and tube wells) are all linked, according to evidence from a bounds-testing study of ARDL [80,81]. Agricultural production increases with the availability of formal loans, new technologies (such as tractors), and an increase in workers. The positive impact of carbon dioxide emissions on agricultural productivity is minimal in both scenarios [82].

## 3 Methodology

### 3.1 Conceptual framework

**Fig. 3:**
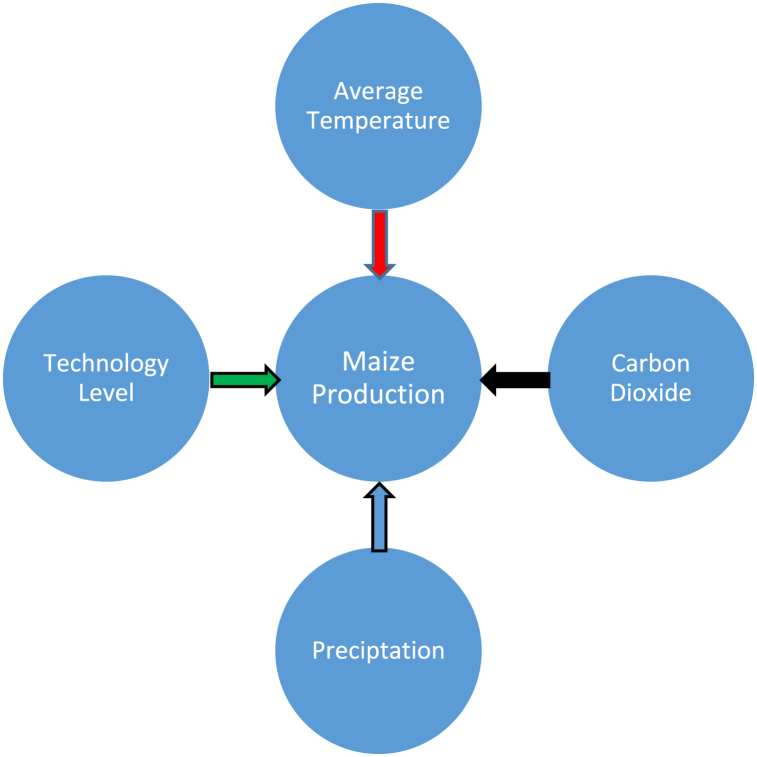
Conceptual framework.

Reviewing the related literature allowed us to create a research framework. The model examines the relationship between the independent variables (average temperature, carbon dioxide, precipitation, and technology level) and the dependent variable (maize production). The conceptual framework is shown in Fig. 3.

### 3.2 Data sample of the study

The primary aim is to find out the effect of climate change on the production of maize in Pakistan, which has been empirically examined using time-series data from 1990 to 2020 that was gathered from the Central Banks of Pakistan websites, World Economic Indicators, and the World Bank. For this purpose, the analyses have been conducted through Eviews-9.

### 3.3 Model description

The application of ARDL models enables the assessment of the influence of previous variable values on subsequent values. Encoding delays and persistence is crucial in modeling social and economic systems. Regarding model selection, the ARDL approach is employed to determine the best lag times to be applied. Researchers can ascertain their model's most suitable lag time by employing information metrics such as the Akaike Information Criterion (AIC) or the Schwarz Bayesian Information Criterion (BIC). The utilization of ARDL models enables the testing of hypotheses and the derivation of inferences regarding the associations among variables. In order to assess the validity of the model, researchers often examine the importance of the coefficients, the goodness of fit, and diagnostic tests [[72], [73], [74],^81^].

Various lag length selection criteria have been presented as potential solutions. These include the Akaike Information Criterion, the Schwarz Information Criterion, the Hannan-Quinn Criterion, the Final Prediction Error, and the Corrected version of AIC. In this study, we employed the Akaike Information Criterion to examine the criteria for selecting lag, including normal errors, non-normal errors, and a structural break. It has been established that the Schwarz Information Criterion (SIC) has superior performance when used in large samples. Additionally, it has been observed that in situations involving regime transitions or shocks to the system, no criterion proves effective in accurately determining the appropriate lag time.

In this investigation, we experimentally explored the relationships between the chosen variables and their dynamic short-term and long-term associations, as the conceptual framework is shown in Fig. 3. The long-term relationship between nonstationary variables was investigated using an autoregressive distributed lag and the Bounds cointegration test. The error term may be eliminated by making all equation variables have the same stationarity rate. Although some variables might be nonstationary, for a linear combination, there is a possibility to be co-integrated. Hence, a set of variables may be co-integrated if a symmetric long-run relationship exists between variables [75]. The time series data necessitates checking for stationarity and cointegration among variables before estimating ADRL. The studies by Pesaran (1997), Shin (1998), and Pesaran and Shin (1998 together), Researchers interested in learning more about panel data causality and time series tests that rely on integration and cointegration results should look at the chapters on those topics in the books by Menegaki (2018) and Fuinhas and Marques (2019), respectively titled "The Economics and the Econometrics of the Energy-Growth Nexus" and "The Extended Energy-Growth Nexus". To find the nexus between the variables equation (1) is constructed as follows:

(1) $$y_{t=C_{0}+}\sum_{k=1}^{p}\beta_{k}y_{t-k}+\sum_{j=0}^{t}a_{j+1}x_{t-j}+u_{t}$$

To measure the long and short run association among the variable, equation (1) is modified to equation (2). Equation (2) can be written in ARDL form are as follows:

(2) $${\Delta \mathrm{Ln}(MZP}_{t)=}\beta_{0}+\beta_{1}L_{n}\left({MZP}_{t-1}\right)+\beta_{2}+\beta_{3}\;\mathrm{Ln}\left({AVEGTP}_{t-1}\right)+\beta_{5}\;\mathrm{Ln}\left({CO2}_{t-i}\right)+\beta_{6}\;\mathrm{Ln}\left({PRPT}_{t-1}\right)+\beta_{7}\;\mathrm{Ln}\left(TWL\right)+\sum_{i=1}^{p}\alpha_{i}\Delta \mathrm{Ln}\left({MZP}_{t-i}\right)+\sum_{l=1}^{s}\alpha_{i}\Delta \mathrm{Ln}\left({AVEGTP}_{t-l}\right)+\sum_{m=1}^{v}\alpha_{i}\Delta \mathrm{Ln}\left({CO2}_{t-m}\right)+\sum_{n=1}^{y}\alpha_{i}\Delta \mathrm{Ln}\left({PERT}_{t-n}\right)+\sum_{0=1}^{w}\alpha_{0}\Delta \mathrm{Ln}\left({TWL}_{t-o}\right)+{\gamma ECT}_{t-1}+u_{t}$$

The variables used in the model are described in Table 1.

**Table 1:** Description of variable (source: World bank and GoP).

Variable	Code	Measurement
***Maize Production***	*MZP*	*Maize Yield in (000) Tones*
***Average Temperature***	*AVGTP*	*Temperature measured in Celsius*
***Precipitation***	*PRPT*	*Precipitation measured in millimeters*
***Carbon Dioxide***	*CO2*	*Carbon dioxide measured by metric tons per capita*
***Tube Well***	*TWO*	*Tube Well Irrigation (million hectares)*

## 4 Results and discussion

### 4.1 Descriptive statistics

The findings of the variables that are descriptive statistics are available in Table 2. This table explains the average value of maize production, Average Temperature (AVEGTP), Carbon Dioxide (CO2), Precipitation (PRCT), and Technology used (TWL) in Pakistan: 3086, 14.082, 27.96, 21.00, 0.697, 298.48, 202251, and 134.09. The highest and lowest values of individual variables explain that MZP has a maximum value of 7883.10 and a lowest value of 1179.30; however, the recorded lowest maximum value is for CO2. The AVEGTP and PRPT are skewed negatively, as shown by the symmetrical distribution of the variables, while another variable is positively skewed. All the variables are normally distributed, as established by the probability value of Jarque Bera.

**Table 2:** Descriptive statistics.

	MZP	AVEGTP	CO2	PRPT	TWL
Mean	*3086*	*21.00*	*0.697*	*298.48*	*7.954*
Median	*2797*	*21.07*	*0.697*	*310.9*	*8.270*
Maximum	*7883*	*21.87*	*0.919*	*392.5*	*8.590*
Minimum	*1179*	*19.82*	*0.506*	*187.5*	*6.410*
Std. Dev.	*1912*	*0.495*	*0.105*	*60.33*	*0.691*
Skewness	*0.839*	*0.413*	*0.193*	*0.133*	*0.957*
Kurtosis	*2.678*	*2.664*	*2.343*	*1.840*	*2.514*
Jarque-Bera	*3.773*	*1.027*	*0.750*	*1.827*	*5.038*
Probability	*0.152*	*0.598*	*0.687*	*0.401*	*0.080*
Sum	*95669*	*650.95*	*21.60*	*9253*	*246.5*
Observations	*31*	*31*	*31*	*31*	*31*

### 4.2 Normality

**Fig. 4:**
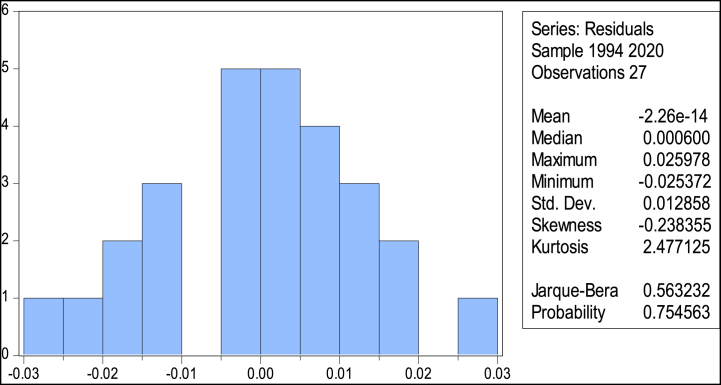
Normality test.

The Jarque-Bera test statistic always gives a positive value; however, if it is not very close to zero, it indicates that the sample data do not adhere to a normal distribution. The value of the test statistic can be found to be 0.563, and its p-value is 0.755. We could not reject the null hypothesis that the data is normally distributed in this scenario shown in Fig. 4.

### 4.3 Unit root test

There are several ways to test whether something is stationary. Consequently, our inquiry utilized widely recognized unit root tests such as the Augmented Dickey-Fuller (ADF) and the Phillips-Perron (PP). Table 3 shows the unit root results of the series at the level and first differences, as well as the constant only and constant with trend specifications used to find stable variables. The results of the Augmented Dickey-Fuller (ADF) and Phillip-Perron (PP) unit root tests show that three variables are stationary. Two are not at the level, and all variables become stationary at the first Difference with constant and constant, with the trend at the 1 %, 5 %, and 10 % significance levels, respectively. The variables are not stationary because they are integrated into order one (1). This emphasizes the need to undertake an ARDL-bound assessment cointegration test to determine whether the variables have a long-term connection or relationship.

**Table 3:** Unit root test.

	Augmented Dickey-Fuller							
Variables	Level				1st Difference			
	C	Sig. Level	C & T	Sig. Level	C	Sig. Level	C & T	Sig. Level
***MZP***	*0.982*	*no*	*0.999*	*no*	*0.018*	**	*0.091*	*
***AVEGTP***	*0.051*	*	*0.738*	*no*	*0.000*	***	*0.000*	***
***CO2***	*0.712*	*no*	*0.003*	***	*0.004*	***	*0.000*	***
***PRPT***	*0.0001*	***	*0.767*	*no*	*0.000*	***	*0.000*	***
***TWL***	*0.039*	**	*0.484*	*no*	*0.739*	*no*	*0.034*	**
***Phillip-Perron (PP) unit root tests***								
***MZP***	*1.232*	*no*	*1.234*	*no*	*0.023*	**	*0.019*	*
***AVEGTP***	*0.193*	*no*	*0.934*	*no*	*0.001*	***	*0.000*	***
***CO2***	*0.982*	*no*	*0.074*	*	*0.008*	***	*0.000*	***
***PRPT***	*0.132*	*no*	*0.894*	*no*	*0.001*	***	*0.000*	***
***TWL***	*0.051*	*no*	*0.789*	*no*	*0.894*	*no*	*0.042*	**

Notes: (*) Significant at 10 %; (**) Significant at 5 %; (***) Significant at the 1, and (no) Not Significant, C represents the constant, C&T represents the constant and trend *MacKinnon (1996) one-sided p-values.

Null Hypothesis: The variable has a unit root.

### 4.4 Correlation

A correlation matrix shows the association between environmental and other factors and maize production in Pakistan. According to the results, all factors positively affect Pakistan's production of maize and agricultural value added. However, compared to average temperature, CO2 emissions, and technology use, precipitation had a weaker correlation with agricultural output. The relationships between the variables are shown in Table 4. The average temperature (AVEGTP) has a 0.567 association with maize production (MPZ). This is the positive correlation in the matrix. The correlation between carbon dioxide (CO2) and maize production (MZP) is 0.725, which is highly positive. The correlation coefficient between precipitation (PRPT) and maize production (MZP) is 0.119. There is a strong positive correlation between tube well irrigation (TWL) and maize production (MZP), with a correlation coefficient 0.747.

**Table 4:** Correlation matrix.

Correlation	MZP	AVEGTP	CO2	PRPT	TWL
***MZP***	*1*				
***AVEGTP***	*0.567*	*1*			
***CO2***	*0.725*	*0.628*	*1*		
***PRPT***	*0.119*	*0.315*	*0.055*	*1*	
***TWL***	*0.747*	*0.664*	*0.781*	*0.018*	*1*

### 4.5 Bound test for cointegration

To examine the long-term impact of Average Temperature (AVEGP), Carbon Dioxide (CO2), Precipitation (PRPT), and Tube Well Irrigation (TWL) on Maize Production (MZP) in Pakistan, a cointegration test employing the bound test approach and the results are shown in Table 5.

**Table 5:** Bound test approach.

Test Statistic	Value	k
*F-statistic*	*4.530*	*4*
*Critical Value Bounds*		
*Significance*	*Lower Bound*	*Upper Bound*
*10 %*	*2.123*	*3.210*
*5 %*	*2.451*	*3.610*
*2.50 %*	*2.750*	*3.990*
*1 %*	*3.150*	*4.430*

The result of the ARDL bound test for cointegration is shown in Table 4. Given the computed F statistic of 4.530 "(F statistic is greater than Upper Bound Value)," the results show no cointegration among variables and rejection of the null hypothesis, indicating a long-run relationship among these variables.

### 4.6 The result of the ARDL model

#### 4.6.1 Short-run impact of climate variables on maize production

The short-run dynamic model describes the convergence rate to the equilibrium following a disturbance to the equation. The model of cointegration of the short run has been devised as the ARDL Framework has the short-run active parameter. Table 6 shows the short-run result: At the 1 % significance level, the average temperature (AVEGTP) has a significant short-run impact on maize production (MZP) (Coefficient = −3.237, p-value = 0.001). According to Ref. [81], average temperature has a short-run impact on maize production, consistent with the current study's findings. Carbon dioxide (CO2) has a significant short-run impact on maize production (MZP) at the 5 % significance level (Coefficient = 0.757, p-value = 0.005). According to [87], carbon dioxide has a short-run impact on maize production, consistent with the current study's findings. At the 10 % significance level, precipitation (PRPT) has a significant short-run impact on maize production (MZP) (coefficient = −0.141, p-value = 0.062). According to [88], precipitation has a short-run impact on maize production, consistent with the current study's results. The short-run impacts of tube well (TWL) on maize production (MZP) are statistically significant at the 1 % level (coefficient = −16.795, p-value = 0.004). The current study's findings are consistent with those of [89], which state that tube wells have a short-run impact on maize production.

**Table 6:** Short-run impact of climate variables on maize production.

Variable	Coefficient	Std. Error	t-Statistic	Prob.	Sig. level
*AVEGTP*	*−3.237*	*0.842*	*−3.843*	*0.001*	***
*CO2*	*0.757*	*0.242*	*3.117*	*0.005*	**
*PRPT*	*−0.141*	*0.072*	*−1.960*	*0.062*	*
*TWL*	*−16.795*	*5.187*	*−3.238*	*0.004*	***
*CointEq(-1)*	*−0.255*	*0.068*	*−3.764*	*0.001*	***

Cointeq = MZP - (−12.6913*AVEGTP + 2.9692*CO2+0.5519*PRPT -0.8538*TWL + 23.1550).

(*) Significant at 10 %; (**) Significant at 5 %; (***) Significant at 1 % and (no) Not Significant.

#### 4.6.2 Long-run impact of climate variables on maize production

Table 7 shows that the long-run ARDL result, the average temperature in the long run (AVEGTP), has a significant impact on maize production (MZP) (Coefficient = −17.449, p-value = 0.013). This was determined at the 5 % significance level. The results of the current study are consistent with those found in a previous study [90; 91], which states that average temperature impacts maize production over the long run. Carbon dioxide (CO2) has a significant impact on maize production (MZP) over the long run (Coefficient = 3.603, p-value = 0.001). This was determined at a 1 % level of significance. The current study's findings are consistent with those of a previous study [92], which concluded that carbon dioxide has a long-run impact on maize production. Precipitation (PRPT) has a long-run impact on maize production (MZP) (coefficient = −1.548, p-value = 0.084). This was measured at the 10 % significance level. A prior study [93] found that precipitation has a long-run impact on maize productivity, supported by the current study. The tube well (TWL) significantly impacts maize production (p-value = 0.021, coefficient = −4.222). This was examined at a 5 % level of significance. This study confirms the previous study's findings[94; 95] that tube wells have a long-term impact on maize production.

**Table 7:** Long-run impact of climate variables on maize production.

Variable	Coefficient	Std. Error	t-Statistics	Prob.	Sig. Level
***AVEGTP***	*−17.449*	*4.723*	*−2.687*	*0.013*	**
***CO2***	*3.603*	*0.734*	*4.044*	*0.001*	***
***PRPT***	*−1.548*	*0.305*	*−1.807*	*0.084*	*
***TWL***	*−4.222*	*1.649*	*−5.517*	*0.021*	**
***C***	*35.128*	*6.611*	*3.502*	*0.002*	***

(*) Significant at 10 %; (**) Significant at 5 %; (***) Significant at 1 % and (no) Not Significant.

### 4.7 FMOLS and DOLS test

The estimated results of the FMOLS and DOLS models are presented in Table 8. These models provide coefficients that can be employed to determine the long-term impact. In the FMOLS and DOLS models for average temperature, precipitation, and tube well, respectively, its coefficient is negative and statistically significant at 10 %, 5 %, and 1 %. Thus, the average temperature, precipitation, and tube well harm the region's long-term economic growth. In both the Fully Modified Ordinary Least Squares (FMOLS) and Dynamic Ordinary Least Squares (DOLS) models, it is shown that only the variable representing carbon dioxide (CO2) exhibits a positive and statistically significant impact at the 10 % level. The sign and significance of the FMOLS and DOLS affirm the long-run estimation of the ARDL model.

**Table 8:** FMLOS and DOLS test.

	*AVGTP*	*PRPT*	CO2	TWL
**FMOLS**	−0.874	−1.243	0.839	−0.109
	(0.034) **	(0.000) ***	(0.092) *	(0.032) **
**DOLS**	−1.032	−0.948	0.729	−1.198
	(0.057) **	(0.004) ***	(0.098) *	(0.056) *

Value in parentheses shows the standard error.

### 4.8 Diagnostic test and model stability

Before starting an economic data analysis, perform diagnostic tests to confirm the estimated model's accuracy. We carried out a series of diagnostic tests to validate the robustness of the model as a final step in output analysis. The Heteroscedasticity test, the Serial correlation test (Brush & Godfray LM test), the Normality test (Jaque-Bera), and the Functional form test (Ramsey's RESET) were among them. As shown in Table 9, diagnostic tests indicate this because long- and short-run estimates are devoid of serial correlation, misspecification of the short-run model, non-normality of the error component, and heteroscedasticity.

**Table 9:** Stability test.

	Chi-statistic	F-statistic
*Breusch-Godfrey Serial Correlation LM Test*	*χ*2(2) = 2.123	*F* = 2.968
	*Prob.=* 0.078	*Prob.=* 0.089
*Breusch-Godfrey Heteroskedasticity Test*	*χ*2(1) = 0.692	*F* = 0.782
	*Prob.=* 0.789	*Prob.=* 0.841
*Jarque-Bera Normality Test*	*χ*2(2) = 0.563	NA
	*Prob.= 0.754*	
*Ramsey RESET Test*	*χ*2(1) = 0.314	*F* = 0.087
	*Prob.=* 0.879	*Prob.=*0.889

#### 4.8.1 Chow breakpoint test

According to the data, the p-value of the f-statistics is 0.0002, which is less than the 5 % threshold of significance; hence, this research rejects the null Hypothesis, indicating a structural change. The following table makes this very evident. The slope and coefficient of the two periods varied according to Table 10 findings.

**Table 10:** Chow breakpoint test: 2008.

Equation Sample: 1990 2020			
F-statistic	8.119	Prob. F (7,17)	0.0002
Log likelihood ratio	45.527	Prob. Chi-Square (7)	00000
*Wald Statistic*	*56.836*	*Prob. Chi-Square (7)*	*00000*

Null: There is no Structural Change.

Alternative: There is Structural Change.

### 4.9 Granger causality test

Determining whether two variables are cointegrated is crucial in evaluating long-term Granger causality using vector error correction [80]. The preceding outcomes of the Granger causality test indicate that financial development is vital in fostering economic growth in Ethiopia. This supports the enduring validity of the "supply-led" expansion hypothesis. The research mentioned above conducted by Ref. [82] has yielded comparable results. Table 11 illustrates that, like the long-term perspective, a unidirectional causality exists from environmental and technological factors to economically sustainable maize production in Pakistan. This data further supports the notion that the expansion of the agricultural sector, in both the short and long term, is being driven by changes in environmental factors and technological advancements.

**Table 11:** Long- and short-run Granger causality test.

	Null Hypothesis	Obs.	Lags	Coefficient	Prob.
**Long-run**	*Average Temperature does not Granger cause Maize Production*	31	1	0.025	0.003***
	*Carbon dioxide does not Granger cause Maize Production*	31	1	0.068	0.0012***
	*Precipitation does not Granger cause Maize Production*	31	1	0.099	0.065**
	*Technology Level does not Granger cause Maize Production*	31	1	0.012	0.802
**Short run**	*Average Temperature does not Granger cause Maize Production*	31	1	4.994	0.076***
	*Carbon dioxide does not Granger cause Maize Production*	31	1	0.046	0.873
	*Precipitation does not Granger cause Maize Production*	31	1	0.055	0.045***
	*Technology Level does not Granger cause Maize Production*	31	1	0.546	0.458

**Notes:** The signs of *, **, and *** indicate the significance levels at 10 %, 5 %, and 1 % to reject the null hypothesis of the direction of causality, respectively.

## 5 Discussion

The results are that average temperature (AVEGTP) has a significant short-term impact on maize production (MZP) at the 1 % significance level (coefficient = −3.237, p-value = 0.001). The current study agrees with [39] that average temperature short-term affects maize production. CO2 significantly impacts maize output (MZP) at 5 % significance (coefficient = 0.757, p-value = 0.005). According to Ref. [83], carbon dioxide has a short-run impact on maize production. Precipitation (PRPT) affects maize output (MZP) in the short run at the 10 % significance level (coefficient = −0.141, p-value = 0.062) [84], which is supported by the current study. Tube well (TWL) short-run impacts on maize production (MZP) are statistically significant at the 1 % level (coefficient = −16.796, p-value = 0.004). This study confirms [ [66,79]] that tube wells have a short-run impact on maize productivity.

According to the long-run ARDL, average temperature (AVEGTP) significantly impacts maize production (MZP) (coefficient = −17.449, p-value = 0.013). This was 5 % significant. This research confirms a previous study [37] that average temperature affects maize production over time. CO2 affects maize production (MZP) (coefficient = 3.603, p-value = 0.001). At 1 % significance, this was determined. This study supports a prior study [78]. In the long run, precipitation impacts maize production (MZP) (coefficient = −1.548, p-value = 0.084) at a 10 % significance level. This investigation confirms a previous study [40]. The tube well (TWL) significantly impacts maize productivity over time (p-value = 0.0206, coefficient = −4.223). The significance level of 5 % was used. This study confirms a prior study [85].

### 5.1 Conclusion

Maize production is essential for sustainable food production, consumption, and security. Because of its large production capacity, nutritional content, range of uses, advantages to soil health, and contributions to economic prosperity, it is an essential crop in producing a resilient and sustainable food system. Inside the ARDL framework, the cointegration model of the short-run active parameter and the short-run effect has been developed. The originality of a study on this topic would depend on the presented empirical findings and implications. For instance, a study that analyzes the impact of climate change on maize production in a specific region or country and offers context-specific adaptation strategies or policy recommendations would be considered original. The efficacy of specific policies may vary across different provinces and areas in Pakistan. To obtain the most current and comprehensive information regarding policies about environmental changes and maize production in Pakistan, it is advisable to consult official government sources, agricultural agencies, and pertinent research groups. By inquiring about any current adjustments or advancements in this domain, one may also inquire about them. Policies promoting conservation agricultural techniques, such as decreased tillage and cover cropping, enhance soil health, erosion management, and water conservation. Sustainable maize production techniques can be disseminated through extension services and farmer training programs. Utilizing these programs can facilitate well-informed decisions on planting, harvesting, and pest management.

### 5.2 Implication

Maize is a staple grain in many parts of the world, but climate change threatens its production. The success of the maize crop is critical to the sustainability of food production, consumption, and food security. Empirical research shows that increasing temperatures and altered rainfall patterns may impair maize yields, particularly in areas without substantial irrigation. There are significant implications for the future of sustainable agriculture. It first highlights the need for farmers to adopt new agricultural practices, switching to drought-resistant maize varieties, and establishing irrigation systems in regions where it is practicable. Second, it emphasizes the need for global efforts to reduce greenhouse gas emissions and address climate change. Carbon emissions must be reduced, and investments in renewable energy and other low-carbon technologies must be made. Third, it underlines the need for policymakers to put sustainable agricultural growth first to preserve food supply and the economy from the consequences of climate change. This area includes R&D for climate-resilient crop and agricultural practice development, aid to smallholder farmers, and advocacy for sustainable land use. Overall, the impact of climate change on maize yield emphasizes the vital need for action to tackle this worldwide challenge and ensure agriculture's long-term survival for future generations.

### 5.3 Future research direction and limitations

This research can shed light on the opportunities and dangers of climate change for maize production and food security in Pakistan and other regions with comparable issues. Researchers could investigate further the findings presented in the research to gain a deeper understanding of this urgent issue. By utilizing robust data, they can develop productive solutions. Examine how the mobility and trade of maize in Pakistan are affected by the abiotic stress brought on by climate change. This study aims to analyze the impacts of climate-induced stress on national and regional food prices, distribution networks, and the availability of healthy food. This study aims to comprehensively analyze the financial implications of various responses to climate-induced abiotic stress on maize productivity. The focus will be on evaluating adaptation and mitigation strategies to assess their possible effects on the financial aspects of maize production. As a result, policymakers will have an enhanced ability to allocate resources more effectively.

## Funding

National Science Fund of China (The impact mechanism and emergency response mechanism of agricultural enterprise performance under major public health emergencies due to high volatility of supply and demand) Grant **No: 72063008**.

## 6 Ethics approval and consent to participate

This is an observational study. We confirmed that no ethical approval is required.

## Consent to participate is not applicable

Consent for publication: Not applicable.

## Data availability

The datasets used and analyzed during the current study are available from the corresponding author upon reasonable request.

## CRediT authorship contribution statement

**Muhammad Rizwanullah:** Conceptualization, Data curation, Formal analysis, Software, Writing – original draft. **Anhua Yang:** Formal analysis, Investigation, Methodology, Software, Writing – review & editing. **Muhammad Nasrullah:** Conceptualization, Data curation, Formal analysis, Software, Writing – original draft. **Xue Zhou:** Data curation, Formal analysis, Investigation, Methodology, Writing – review & editing. **Atif Rahim:** Data curation, Formal analysis, Investigation, Methodology, Writing – review & editing.

## Declaration of competing interest

The authors declare that they have no known competing financial interests or personal relationships that could have appeared to influence the work reported in this paper.
